# PRMT5 Inhibition as a Potential Strategy for KRAS Mutant CRC: Downstream Mediators of the PRMT5–KRAS Crosstalk

**DOI:** 10.3390/cimb47080665

**Published:** 2025-08-18

**Authors:** Mark Spivak, Moshe Pahmer, Dorna Delrahimnia, Tzuriel Sapir, David Shifteh

**Affiliations:** 1 College of Medicine, SUNY Downstate Health Sciences University, Brooklyn, NY 11203, USA; mark.spivak@downstate.edu; 2 Department of Biology, Yeshiva College, Yeshiva University, New York, NY 10033, USA; mpahmer@mail.yu.edu; 3 College of Health Professions, Pace University, New York, NY 10038, USA; dd46464n@pace.edu; 4 Perelman School of Medicine, University of Pennsylvania, Philadelphia, PA 19104, USA

**Keywords:** PRMT5, KRAS, CRC, MYC, E2F1, EIF4E

## Abstract

Colorectal cancer (CRC) is a leading cause of cancer-related mortality worldwide with KRAS mutations present in nearly 45% of cases. Compared to KRAS wild-type (WT) CRC, KRAS-mutant CRC is associated with poorer prognosis and fewer effective treatment options. Protein Arginine Methyltransferase 5 (PRMT5), an epigenetic regulator involved in diverse cellular processes, is currently under investigation as a therapeutic target in multiple cancer types. Our previous work demonstrated that PRMT5 inhibition produces stronger therapeutic effects in KRAS-mutant CRC cells than in KRAS WT cells, suggesting potential crosstalk between PRMT5 and KRAS. In this study, we aimed to identify key downstream proteins that may mediate this interaction. Through a literature review, protein–protein interaction analysis (STRING database), gene expression analysis (GEPIA database), and correlation analysis (GEPIA database), we identified MYC, E2F1, and EIF4E as critical candidates. These proteins are shown to interact with both PRMT5 and KRAS in STRING, are overexpressed in CRC tumor samples, and show positive gene expression correlations with PRMT5 and KRAS in patient data. These findings are significant, as they provide new insights into the PRMT5–KRAS crosstalk and suggest potential targets for novel and combination therapies in KRAS-mutant CRC. Further research and biological experiments are needed to verify and outline the exact molecular processes behind MYC, E2F1, and EIF4E’s interactions with both PRMT5 and *KRAS*.

## 1. Introduction

Colorectal cancer (CRC) holds the third highest incidence, and third highest number of deaths, among all other cancers for men and women in the United States [1]. While fewer than 20% of metastatic CRC patients survive past five years from diagnosis, an increased use of tumor-specific molecular characterization, and thus treatment plan tailoring, is improving overall survival [2]. Elucidating the relationship between key cell growth modulators is an important first step in such treatment plan tailoring, as it can provide clues as to which treatments may work. For example, understanding that KRAS and BRAF are both downstream of EGFR explains why the EGFR inhibitor cetuximab is ineffective in KRAS-mutated CRC and why using cetuximab in combination with the BRAF inhibitor encorafenib is now FDA-approved therapy for patients with a previously treated V600E BRAF mutated CRC [3,4,5,6].

The vast range of cellular interactions in which Protein Arginine Methyltransferase 5 (PRMT5) partakes, therefore, is of peak interest. PRMT5 is a type II methyltransferase which, along with its cofactor S-Adenosyl methionine (AdoMet/SAM), methylates arginine residues on histone and non-histone proteins to catalyze the formation of symmetric dimethylarginine derivative (SDMA) or monomethylated arginine derivative (MMA) [7,8]. PRMT5 activity leads to chromatin restructuring as well as transcriptional control and the regulation of gene expression within the cell [7,9]. Because of PRMT5’s key role in histone modification and in modulating the activity of several transcription factors and growth pathway signals, awry PRMT5 activity can prove pivotal in the cell’s progression toward an oncogenic state. Indeed, PRMT5 is overexpressed in several cancers including melanoma, multiple myeloma, glioblastoma, lung, bladder urothelial, gastric, ovarian, and colorectal cancers, and PRMT5 expression correlates with poor patient prognosis [7,10,11,12,13,14,15,16,17,18,19]. PRMT5 inhibition and knockdown dramatically reduce cellular proliferation, migration, and colony-forming abilities, and they increase apoptosis and cell-cycle arrest both in in vitro and in vivo studies [20,21]. PRMT5 is currently being tested as a potential therapeutic target in several cancer types, and PRMT5 inhibition is currently in clinical trials [22,23,24,25].

Of particular interest, previous work from our group has indicated that PRMT5 interacts with KRAS, an upstream activator of both the ERK1/2 and PI3K pathways [26]. KRAS mutations are found in nearly 45% of CRCs and act as a molecular switch to constitutively stimulate cellular growth, survival, and proliferation, leading to tumorigenesis [27]. Yet, despite decades of intense research, a selective inhibitor for KRAS is yet to be developed. Patients with KRAS mutant CRC, therefore, experience poor outcomes and a worse prognosis [28]. Our group’s previous work showed that KRAS-mutant CRC cells show greater response to PRMT5 inhibition treatment compared to KRAS-WT CRC cells [26]. The relationship between KRAS and PRMT5, therefore, sheds light on a critically important conclusion: the apparent susceptibility of KRAS-mutant CRC cells to PRMT5 inhibition treatment may be used to treat KRAS-mutant CRC in the clinical setting.

Thus, in this study, we sought to determine and shed some light onto the possible mechanisms of interaction between KRAS and PRMT5. We first conducted a literature review to determine which key downstream proteins may be involved in mediating the potential crosstalk between PRMT5 and KRAS. We next used the STRING database to determine which of the key downstream proteins that we determined from our literature review have demonstrated interactions with PRMT5 and KRAS. We then used the Gene Expression Profiling Interactive Analysis (GEPIA) database to determine which of the remaining proteins are overexpressed in CRC patient tumor samples compared to normal colon and rectum patient samples. Finally, we used the GEPIA database to determine which of the remaining proteins have an expressional correlation with PRMT5 and KRAS in CRC patient tumor samples. We therefore propose three possible downstream mediators of the PRMT5–KRAS crosstalk for the first time as a possible molecular mechanism for the greater efficacy of PRMT5 inhibition in KRAS mutant CRC when compared to KRAS WT CRC.

## 2. Materials and Methods

### 2.1. STRING Database

The STRING database was used to determine which of the key downstream proteins that we determined from our literature review have demonstrated interactions with PRMT5 and KRAS [29]. This was performed by logging onto the STRING database, selecting multiple proteins, listing all of the determined protein names, selecting Homo sapiens for the organism, and then clicking search. This generated a diagram for all of the determined interactions between the listed proteins.

### 2.2. Gene Expression Profiling Interactive Analysis (GEPIA): Gene Expression

The GEPIA database was used to analyze the RNA-Seq data of CRC patients from The Cancer Genome Atlas (TCGA) database to determine which of the remaining key downstream proteins are overexpressed in colon and rectum patient tumor samples [30]. This was performed by logging onto the GEPIA database and selecting the Single Gene Analysis tab and inserting each protein. Next, the Expression DIY Profile tab was then selected to arrive at Gene Expression Profile. We then inputted the desired protein options and clicked plot, and a plot of the gene expression for all proteins was generated.

### 2.3. Gene Expression Profiling Interactive Analysis (GEPIA): Correlation Analysis

The GEPIA database was used to analyze the RNA-Seq data of CRC patients from The Cancer Genome Atlas (TCGA) database to determine which of the remaining key downstream proteins are positively correlated with PRMT5 and KRAS in colon and rectum patient tumor samples. This was performed by logging onto the GEPIA database and selecting the Multiple Gene Analysis tab and then selecting Correlation Analysis. We then inputted the desired protein options and clicked plot, and a plot of the correlation analysis for all of the proteins was generated.

## 3. Results

### 3.1. Key Downstream Proteins That May Be Involved in Mediating the Potential Crosstalk Between PRMT5 and KRAS

**Figure 1:**
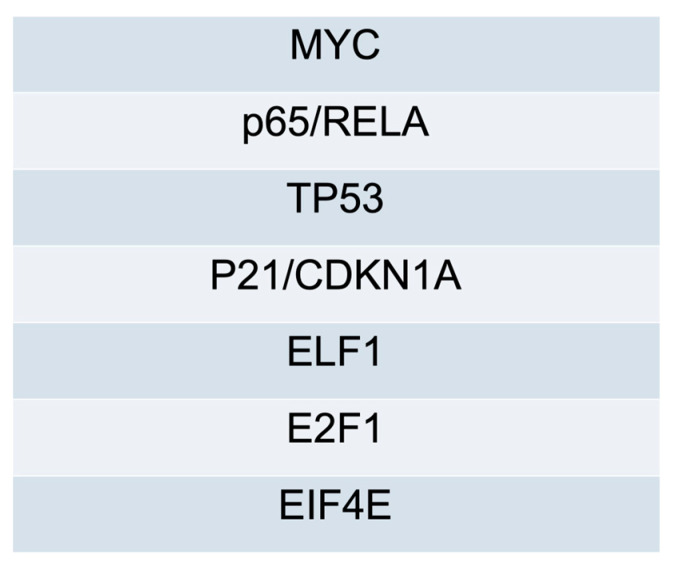
MYC, p65/RELA, TP53, P21/CDKN1A, ELF1, E2F1, and EIF4E may be the key downstream proteins involved in mediating the potential crosstalk between PRMT5 and KRAS based on a literature review.

Our group’s previous work showed that KRAS-mutant CRC cells show greater response to PRMT5 inhibition treatment compared to KRAS-WT CRC cells [26]. In this study, we therefore sought to determine and shed some light onto the possible mechanisms of interaction between KRAS and PRMT5. As such, we first conducted a literature review to determine which key downstream proteins may be involved in mediating the potential crosstalk between PRMT5 and KRAS. Through our literature review, we identified seven key downstream proteins, MYC, p65/RELA, TP53, P21/CDKN1A, ELF1, E2F1, and EIF4E, that may be involved in mediating the potential crosstalk between PRMT5 and KRAS. These key downstream proteins can be seen in Figure 1 below.

### 3.2. Key Downstream Proteins That Interact with PRMT5 and KRAS in the STRING Database

**Figure 2:**
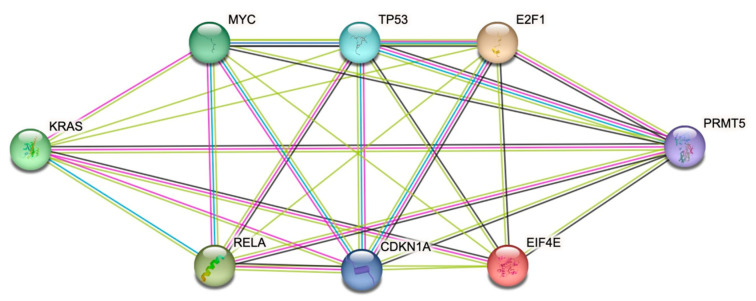
MYC, p65/RELA, TP53, P21/CDKN1A, E2F1, and EIF4E have been shown to interact with PRMT5 and KRAS in the STRING database with an interaction score > 0.150.

After identifying that MYC, p65/RELA, TP53, P21/CDKN1A, ELF1, E2F1, and EIF4E may be the key downstream proteins involved in mediating the potential crosstalk between PRMT5 and KRAS based on our literature review, we next used the STRING database to determine which of the key downstream proteins stated above that we determined from our literature review have demonstrated interactions with PRMT5 and KRAS. Through our generated STRING database diagram, we identified that six of the key downstream proteins, MYC, p65/RELA, TP53, P21/CDKN1A, E2F1, and EIF4E, have demonstrated interactions with PRMT5 and KRAS in the STRING database with an interaction score > 0.150. These key downstream proteins can be seen in Figure 2 below.

### 3.3. MYC, TP53, E2F1, and EIF4E Are Overexpressed in Colon and Rectum Patient Tumor Samples

**Figure 3:**
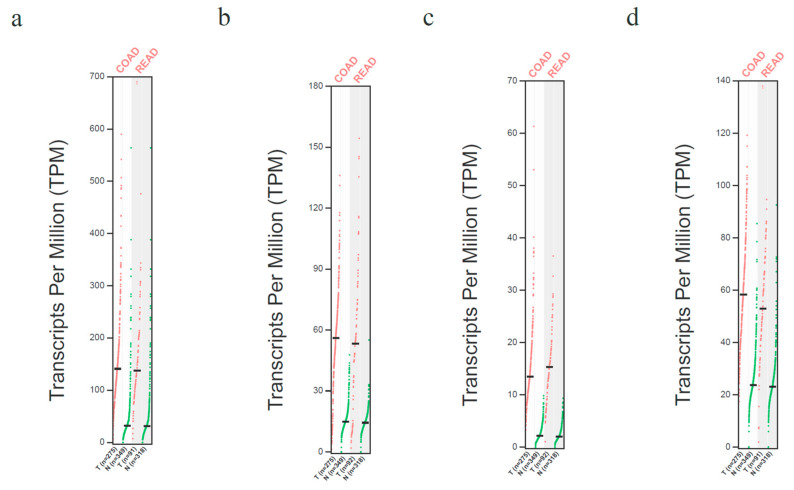
MYC, TP53, E2F1, and EIF4E are shown to be overexpressed in colon and rectum patient tumor samples. (**a**) MYC is 3 + Fold overexpressed in colon and rectum patient tumor samples compared to normal colon and rectum patient samples (q < 0.01). (**b**) TP53 is 3 + Fold overexpressed in colon and rectum patient tumor samples compared to normal colon and rectum patient samples (q < 0.01). (**c**) E2F1 is 3 + Fold overexpressed in colon and rectum patient tumor samples compared to normal colon and rectum patient samples (q < 0.01). (**d**) EIF4E is 2 + Fold overexpressed in colon and rectum patient tumor samples compared to normal colon and rectum patient samples (q < 0.01).

After identifying that MYC, p65/RELA, TP53, P21/CDKN1A, E2F1, and EIF4E have been shown to interact with PRMT5 and KRAS in the STRING database, we then used the Gene Expression Profiling Interactive Analysis (GEPIA) database to determine which of the remaining proteins are overexpressed in CRC patient tumor samples compared to normal colon and rectum patient samples. Through our generated GEPIA database plot, we identified that four of the key downstream proteins, MYC, TP53, E2F1, and EIF4E, are overexpressed in colon and rectum patient tumor samples (2 + Fold overexpressed with q < 0.01). These key downstream proteins can be seen in Figure 3 below.

### 3.4. E2F1, EIF4E, and MYC Are Positively Correlated with PRMT5 and KRAS in Colon and Rectum Patient Tumor Samples

**Figure 4:**
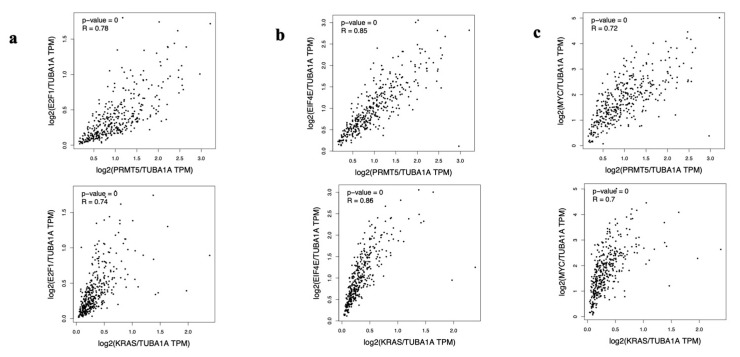
E2F1, EIF4E, and MYC are shown to be positively correlated with PRMT5 and KRAS in colon and rectum patient tumor samples. (**a**) E2F1 gene expression is positively correlated with PRMT5 (*p* < 0.01, R = 0.78) and KRAS (*p* < 0.01, R = 0.74) gene expression in colon and rectum patient tumor samples. (**b**) EIF4E gene expression is positively correlated with PRMT5 (*p* < 0.01, R = 0.85) and KRAS (*p* < 0.01, R = 0.86) gene expression in colon and rectum patient tumor samples. (**c**) MYC gene expression is positively correlated with PRMT5 (*p* < 0.01, R = 0.72) and KRAS (*p* < 0.01, R = 0.7) gene expression in colon and rectum patient tumor samples.

After identifying that MYC, TP53, E2F1, and EIF4E are shown to be overexpressed in colon and rectum patient tumor samples in the GEPIA database, we then used the GEPIA database to determine which of the remaining proteins have an expressional correlation with PRMT5 and KRAS in CRC patient tumor samples. Through our generated GEPIA database plot, we identified that three of the key downstream proteins, E2F1, EIF4E, and MYC, are positively correlated with PRMT5 and KRAS in colon and rectum patient tumor samples (*p* < 0.01, R > 0.7). These key downstream proteins can be seen in Figure 4 below.

## 4. Discussion

Despite decades of research, there are still no effective targeted treatments for the nearly 45% of colorectal cancer (CRC) patients harboring a mutation in their KRAS gene. Protein Arginine Methyltransferase 5 (PRMT5) is an epigenetic regulator undergoing clinical trials as a potential therapeutic target for cancer.

Our group has previously reported that PRMT5 is overexpressed in *KRAS-*mutant CRC when compared to *KRAS* wild-type (WT) CRC, and that PRMT5 inhibition exhibited greater therapeutic efficacy in *KRAS-*mutant CRC when compared to *KRAS* WT CRC. We therefore proposed that PRMT5 may be a strong therapeutic target for *KRAS-*mutant CRC, and that PRMT5 and *KRAS* may crosstalk. In this study, we investigated several key downstream signal transduction proteins that may be involved in mediating the potential crosstalk between PRMT5 and *KRAS*. This is important, as these key intermediate proteins may be strong therapeutic targets for inhibition as well as valuable therapeutic targets for combination therapy along with PRMT5 inhibitors.

We first conducted a literature review to determine which key downstream proteins may be involved in mediating the potential crosstalk between PRMT5 and *KRAS*. Our initial findings indicated that MYC, p65/RELA, TP53, P21/CDKN1A, ELF1, E2F1, and EIF4E may be key downstream proteins involved in mediating the potential crosstalk between PRMT5 and *KRAS*. All of these proteins, including TP53 and p65, have demonstrated interactions in the literature with PRMT5 and KRAS and are great candidates for further research. We next used the STRING database to determine which of the aforementioned proteins have demonstrated interactions with PRMT5 and *KRAS*. We found that PRMT5 and *KRAS* both interact with MYC, p65/RELA, TP53, P21/CDKN1A, E2F1, and EIF4E with an interaction score > 0.150.

We then used the Gene Expression Profiling Interactive Analysis (GEPIA) database to analyze the RNA-Seq data of CRC patients from The Cancer Genome Atlas (TCGA) database. We first used the GEPIA database to determine which proteins are overexpressed in CRC patient tumor samples compared to normal colon and rectum patient samples. We observed that MYC, TP53, E2F1, and EIF4E are all over 2-fold overexpressed in CRC patient tumor samples compared to normal colon and rectum patient samples (q < 0.01). We next used the GEPIA database to determine which proteins have an expressional correlation with PRMT5 and *KRAS* in CRC patient tumor samples. We found that MYC, E2F1, and EIF4E are all positively correlated with PRMT5 and *KRAS* with an R-value > 0.70 and a *p*-value < 0.01 in CRC patient tumor samples.

Our study thus showcases the key downstream signal transduction proteins that may be involved in mediating the potential crosstalk between PRMT5 and *KRAS*. After running the STRING database analysis, the GEPIA overexpression analysis, and the GEPIA correlation analysis, we identified that MYC, E2F1, and EIF4E are the three finalist candidates that may be involved in mediating the potential crosstalk between PRMT5 and *KRAS*. In fact, these three proteins—MYC, E2F1, and EIF4E—have been extensively documented in previous studies to be downstream mediators of KRAS-driven oncogenesis [31,32,33]. These findings are significant, as these key intermediate proteins may be strong therapeutic targets for inhibition as well as strong therapeutic targets for a PRMT5 combination therapy. In addition, these findings also provide new insights into the PRMT5–KRAS crosstalk and highlight potential avenues for developing new therapies and combination therapies for KRAS-mutated CRC patients. However, as our study is bioinformatic in nature, which has its limitations—including not distinguishing the data by KRAS mutant and WT status—further research and biological experiments are needed to verify and outline the exact molecular processes behind MYC, E2F1, and EIF4E’s interactions with both PRMT5 and *KRAS*.

## 5. Conclusions

In conclusion, after our group’s previous research demonstrated that PRMT5 inhibition shows greater therapeutic effects in KRAS-mutant CRC cells when compared to KRAS WT CRC cells, and that PRMT5 and KRAS may crosstalk, our group sought to determine which key downstream proteins may be mediators of this PRMT5–KRAS crosstalk. After conducting a literature review, a protein interaction analysis using the STRING database, a gene expression analysis using the GEPIA database, as well as a correlation analysis using the GEPIA database, we identified three critical proteins—MYC, E2F1, and EIF4E—that have been shown to have interactions with PRMT5 and KRAS in the STRING database, overexpressed gene expression in CRC patient tumor samples, as well as positively correlated gene expression with PRMT5 and KRAS in CRC patient tumor samples. These findings are significant, as they provide new insights into the PRMT5–KRAS crosstalk and highlight potential avenues for developing new therapies and combination therapies for KRAS-mutated CRC patients.

## Data Availability

The raw data presented and analyzed in this study can be obtained from the GEPIA and STRING databases as explained in the Section 2 above.

## Abbreviations

PRMT5	Protein arginine methyltransferase 5
CRC	Colorectal cancer
COAD	Colon adenocarcinoma
MYC	MYC proto-oncogene, bHLH transcription factor
KRAS	Kirsten rat sarcoma viral oncogene homolog
READ	Rectum adenocarcinoma
GEPIA	Gene Expression Profiling Interactive Analysis
